# Higher education retention in Ireland and Scotland: the role of admissions policies

**DOI:** 10.1007/s10734-024-01259-1

**Published:** 2024-07-11

**Authors:** Cristina Iannelli, Patricia McMullin, Emer Smyth

**Affiliations:** 1 Moray House School of Education and Sport, University of Edinburgh, Edinburgh, EH8 8AQ UK; 2 Department of Social Research, University of Turku, 20014 Turku, Finland; 3 The Economic and Social Research Institute, Dublin, D02 K138 Ireland

**Keywords:** Higher education, Drop-out, School subjects, Social inequalities, Ireland, Scotland

## Abstract

This article provides new insights into the role played by higher education (HE) selection policies in influencing student retention by exploring drop-out patterns in Ireland and Scotland. The Irish and Scottish HE systems differ in relation to the emphasis placed on grades and subjects studied at school and to the degree of autonomy enjoyed by HE institutions in the student admissions process. We investigate whether these system differences matter for student retention using administrative data from national student records and logistic regression modelling. The findings show that in Ireland, HE students have higher chances of dropping out than in Scotland and there are inequalities in these chances among students from different social backgrounds. Moreover, the association between subject matching (but also school grades) and the chances of dropping-out is stronger in Ireland than in Scotland. We conclude that the tighter student selection criteria in Scotland improve retention and reduce social inequalities in drop-out rates. Moreover, admission criteria are found to be important not only to explain between-country differences in drop-out rates but also within-country differences among different HE institutions.

**Supplementary Information:**

The online version contains supplementary material available at 10.1007/s10734-024-01259-1.

## Introduction

The phenomenon of drop-out from higher education (HE) has attracted a good deal of research and policy interest internationally (European Commission, 2015). Understanding this issue is important for at least two reasons: to reduce inequalities in education and the labour market and to improve institutional efficiency. From an equality perspective, high drop-out rates are likely to exacerbate existing inequalities and frustrate efforts to widen access to HE. This is because existing evidence has shown that students from more disadvantaged backgrounds are more likely to drop-out than other students (see next section). From an institutional perspective, when students do not complete their programme of study, there is an inefficient use of staff time and resources and a loss of economic revenue from student fees. There may also be a reputational cost for the HE institution if drop-out rates are associated with their quality of teaching and student support provided.

Across 23 OECD countries, on average only 39% of full-time students graduate from a bachelor’s programme on time (measured by the theoretical duration of the programme) and 67% after three additional years (OECD, 2019). However, there are large country differences in these rates, with the UK and Ireland having the highest completion rates 72% and 63% (and 85% and 81% after three additional years) respectively and Austria, Slovenia and Chile the lowest. Despite large variations across countries in the level and patterning of HE drop-out, most research has been conducted at national or institution level. Moreover, this research tends to focus on the individual and family background characteristics of those who leave HE before completion or on the characteristics of the HE institution entered (Aina et al., 2022), while much less attention has been paid to macro-level factors linked to national HE systems and structures in shaping students’ trajectories in HE (Haas & Hadjar, 2020).

The aim of this paper is to fill this gap. Our starting point is the acknowledgement that individuals’ actions and decisions can be better understood within the wider context in which they are embedded, not only with reference to a particular institution or local context, but also to national institutional structures and policies. More specifically, our paper contributes to the existing literature on HE retention by investigating two national features of the HE system: the role of admission policies and of curriculum continuity between school and HE learning in influencing the level and patterning of course drop-out. Thus, by comparing the HE systems of Ireland and Scotland, we aim to provide important evidence on the macro-micro links which shape students’ educational opportunities (and, in turn, affect their labour market prospects).

Broadly, HE systems can be categorised into those that admit all candidates with the relevant school leaving certificate (such as the *Abitur* in Germany, *Diploma di Maturità* in Italy and *Baccalauréat* in France) and those that select candidates on the basis of their upper secondary grades and/or subjects, sometimes in conjunction with other criteria such as personal statements and interview performance (Haj et al., 2018). While it has proven difficult to accurately measure cross-national variation in rates of non-retention, evidence suggests that systems (such as the Italian system) where HE is open to all in possession of the relevant upper secondary qualification generally have higher rates of tertiary drop-out (van Stolk et al., 2007; European Commission, 2015). Moreover, systems where HE institutions have a high degree of autonomy in selecting students have been found to have higher student completion rates (Orr et al., 2017). Thus, although rarely studied, there are good reasons to believe that the type of criteria used by HE systems for student admission may influence how students fare on entry to HE.

Both the Irish and the Scottish (UK) systems select applicants based on their school grades achieved in the final exams at the end of upper secondary education (*Highers* in Scotland and *Leaving Certificate* in Ireland). However, there are two important differences between these two countries: in Ireland the HE admission system is highly centralised while in Scotland (as in the rest of the UK) applicants are selected at institution (university) level. Moreover, subjects studied at school is a more influential selection criterion (in particular, for entering research-intensive institutions) in Scotland than in Ireland. Thus, in Scotland students are not only selected based on their academic ability but also based on their prior knowledge in the subjects relevant to the field of study an applicant wants to study at university. We expect this feature, together with the greater autonomy that HE institutions have in Scotland, to improve retention and also to reduce social inequalities in the chances of dropping-out in Scotland.

Using rich student record data, we address the following research questions:

Does having studied school subjects related to the field of study taken in HE (i.e. subject matching) enhance retention in HE courses in the two countries? If so, is this relationship stronger in Ireland than in Scotland?

Are social inequalities in the chances of dropping-out higher in Ireland than in Scotland? Can these inequalities be explained by subject matching (i.e. having taken relevant subjects at secondary level) to a larger extent in Ireland than in Scotland?

## Theoretical explanations and empirical evidence on higher education retention

### Individual and institutional factors

Most theoretical explanations and empirical research on HE drop-out focus on micro-level factors linked to students’ characteristics and decision-making, and/or meso-level institutional factors (such as the size of HE institutions and the services they provide) (Aina et al., 2022; Haas & Hadjar, 2019).

The first set of studies point out that leaving a HE course may be a voluntary act or prompted by failing a set of exams (Tinto, 1982), though most existing research considers the two processes in tandem. Non-completers themselves tend to attribute their decision to leave HE to having taken the ‘wrong’ course as well as to financial difficulties and personal problems (including health and family issues) (Davies & Elias, 2003; Moore-Cherry et al., 2015; Yorke & Longden, 2008).

Research has shown higher rates of non-completion among men than women across very different national settings (Aina, 2013; Hovdhaugen, 2009; Kadar-Satat et al., 2016; Liston et al., 2018), though there has been little focus on potential explanations for this pattern.

A number of studies have indicated that *family background* is significantly associated with the chances of university drop-out, with higher rates found among those from working-class or less educated families (Aina, 2013; Contini, Cugnata & Scagni, 2018; Hovdhaugen, 2009; Johnes & McNabb, 2004; Powdthavee & Vignoles, 2009) and households with lower incomes (Bozick, 2007). Several qualitative studies attribute this pattern to the mismatch between the individual class culture of working-class students and the institutional habitus of the university, leading to feelings of marginalisation or not ‘fitting in’ among non-traditional students (Lehmann, 2007; Reay, Crozier & Clayton, 2010). The institutional habitus of a university can be embedded in social networks but also in concrete practices such as the approach to teaching, learning and assessment, thus enhancing progression among already advantaged groups (Thomas, 2002). In this literature, individual and institutional factors reinforce each other in the reproduction of social inequalities in education.

Other studies focus more specifically on the role of *institutional characteristics* in affecting students’ success in HE. Some highlight the importance of institutional commitment to fostering student engagement (Thomas, 2012) and academic and social integration (Tinto, 1975; Thomas, 2012; Georg, 2009). Others point out the importance of services provided by HE institutions to students for their study success (Chen, 2012).

Structural institutional factors, such as the size of institutions, their degree of selectivity, and the composition of the student body, have also been found to contribute to explaining students’ persistence in HE (Kuh et al, 2006). A number of studies in Ireland and Scotland have pointed to the role of institutional characteristics, i.e. types of HE institution and fields of study, in shaping levels of student dropout (Kadar-Satat et al., 2016; Liston et al., 2018; McCoy & Byrne, 2017). In line with other studies, they have found that students attending more selective (research-intensive) institutions have lower dropout rates than students attending other HE institutions. Moreover, students in STEM subjects are more likely to drop-out than students in social sciences, art and humanities. These findings suggest an important role of pre-HE learning in facilitating or hindering students’ HE academic success.

### The link between school learning and HE retention

Upper secondary educational experience is likely to influence adjustment to, and persistence within, HE in two ways. Firstly, higher school grades will be likely to better equip entrants to cope with more challenging course material and therefore will fare better in tertiary assessments. Secondly, prior subject-specific knowledge will provide the foundational skills needed to make progress in a particular field of study.

Much of the literature on HE retention has focused on *college readiness*, though studies have differed in how they define preparedness for HE. It should be noted that the term college readiness can be problematic by attributing any difficulties in adjustment to HE to the individual rather than taking account of the necessity for curriculum and teaching to be responsive to the starting point of individual students (Naylor & Mifsud, 2020; Thomas, 2008). Prior academic achievement at upper secondary level has been found to be highly predictive of the likelihood of HE retention (Cabrera et al., 2013; Liston et al., 2018; Powdthavee & Vignoles, 2009), with one study highlighting the stronger role of prior achievement for less socio-economically disadvantaged groups (DeAngelo & Franke, 2016).

Prior achievement has also been found to play a role in explaining, at least in part, the higher rates of non-completion evident among working-class students in the UK and Ireland (Powdthavee & Vignoles, 2009; Kadar-Satat et al., 2016; McCoy & Byrne, 2017). Moreover, Delaney and Devereux (2020) found that those with higher upper secondary grades tend to obtain ‘better’ degrees (first- or second-class honours), so at least some non-completion will be related to the fact that those with poorer secondary grades are more likely to fail their HE exams. However, even before they reach the exam stage, those with lower levels of prior achievement may struggle to keep up in classes where they are taught alongside those with higher grades.

In contrast to prior achievement, there has been much less focus on the role of the *type of secondary courses* taken in shaping HE adjustment, though studies in the US indicate that having taken more advanced high school placement tracks enhances college retention (Warburton et al., 2001). Other studies in the UK (but also in other countries) have specifically looked at the importance of mathematical preparedness for students’ success in undergraduate mathematics and science programmes, pointing out the existence of a gap between the mathematics skills acquired at school and those required at university (for a summary of this literature see McAlinden & Noyes, 2019). However, none of these studies provide a comprehensive analysis of the link between having studied different school subjects, more or less relevant to the field of study entered in HE, and HE retention.

An exception to this neglect of the role of type of school subject is a study by Smith and Naylor (2001) which suggests that students who are ‘poorly prepared’ (that is, who do not have two related subjects in their prior qualifications) are significantly more likely to drop out of science and social science courses in the ‘old’ (i.e. more selective) UK universities but this is not the case in language or literature courses. This study suggests that, at least in some fields of study, prior access to the relevant specialist knowledge base enhances adjustment to, and retention in, HE courses.

The current article builds on Smith and Naylor’s work (2001) in three ways. Firstly, it looks at subject match across a broader range of course categories and includes, by way of contrast, analysis of retention patterns in courses which have no related subjects at secondary level. Secondly, it explores all types of HE institution, not just more selective universities. It may be that lecturers in less selective institutions adjust course material to allow for less specialist knowledge among students on entry, thus leading to differential effects of subject matching in these settings. Thirdly, it adopts a comparative perspective, contrasting HE systems which place differential emphases on grades and subjects as a basis for admission. The following section describes the HE structure and entry processes in Ireland and Scotland as context for the later analyses.

## Higher education in Ireland and Scotland

There are two main types of institutions within HE in Ireland: seven universities and fourteen institutes of technology.^1^ From the late 1960s, Regional Technical Colleges were set up to offer sub-degree courses in technical areas and were intended to cater for regional labour markets. Over the period 1992 to 2006, these colleges were re-designated as Institutes of Technology, offering degree and postgraduate (including doctoral) degree courses across a range of disciplines. In 2011/2012, the period to which the current analyses relate, 53% of entrants to HE attended a university while 47% attended an institute of technology. HE entry is determined largely by grades in the Leaving Certificate (upper secondary) exam. Applications for undergraduate degree and sub-degree courses in the universities and institutes of technology are centralised nationally through the Central Applications Office (CAO), with students allowed to specify up to ten choices (in rank order) for degree courses and ten for sub-degree courses. Students are allocated ‘points’ on the basis of the grades received and level taken in the exams, with the results for the ‘best’ six subjects used to calculate the final total point score. Certain subjects are required for entry into some fields of study, but requirements are not specified very tightly — for example, having studied any science subject (including Biology) allows applicants to enter any science degree. The points required for courses reflect the number of places offered and the grade profile of applicants, so the requirements for accessing particular courses vary across institutions and from year to year.

In Scotland, HE institutions comprise ancient, old and new universities (former central institutions, equivalent to the polytechnics in England), which differ in status and selectivity.^2^ As in Ireland, students in Scotland must submit a single online application via the Universities and Colleges Admissions Service (UCAS) specifying a list of up to five courses and the institutions providing them for which they are applying. The application is then forwarded by UCAS to the individual institutions which decide whether to make an offer of a place. There are no statutory requirements in terms of number and type of subjects for gaining access to HE institutions but universities expect prospective university candidates to have taken between three and five Highers in their fifth or sixth year of secondary schooling. Universities, particularly the research-intensive universities (the Russell Group universities) in Scotland, as in the wider UK, take secondary school subjects and grades achieved within these subjects into account in their decision to make an offer. Since 2011 the Russell Universities publish information (recently through an interactive online tool)^3^ on the importance of subject choice for progression to university. Eight subjects — English, Maths, Languages, Physics, Chemistry, Biology, Geography, and History — are identified as ‘facilitating’ access to their institutions.

On the basis of these system-level differences, we expect patterns of retention to differ in the following ways:

H1. Overall levels of drop-out will be lower in Scotland than in Ireland because of the greater subject-specific preparedness required in Scotland.

H2. Prior exposure to related subjects will enhance completion rates in Ireland but this pattern will be less evident in Scotland. This is because, in this latter country, the cohort is already highly matched as a result of selection into HE.

H3. Social inequalities in the chances of dropping out will be lower in Scotland than in Ireland. Differences in attainment and school subjects studied by students from different social origins prior to entering HE are likely to be small in Scotland due to the tight entry requirements there and this is expected to reduce social inequalities.

## Data and methods

### Data

For the purpose of our analysis, we use the Higher Education Authority (HEA) student record database for 2011/2012 in Ireland. Data were collected through the Student Record System (SRS) developed by the HEA and Leaving Certificate examination data from the Central Applications Office (CAO) database were matched to these records. The database contains an individual record for each student in each academic year and pertains to all students in HEA-funded institutions, thus excluding private colleges. In the Irish case the database is restricted to new entrants to HE on a full-time undergraduate degree course. New entrants are defined as students entering HE for the first time, so they should have no prior HE experience. For comparability with the Scottish data (see below), the sample used is confined to those aged 21 years or under; this age restriction excludes 18% of new entrants.

We use the Higher Education Statistics Agency (HESA) student record for the academic year 2012/2013 in Scotland. The student record was collected in respect of all students registered in the reporting institution; here we limit the sample to those on full-time undergraduate degree courses. Information on social origin and prior performance were collected by UCAS and were linked to the student records. Because of the absence of certain information (such as social class background) for older entrants, our sample is limited to all full-time first year students aged 21 or under on entry who are new entrants to HE-level courses.^4^

The focus on subject matching has further implications for the sample used in the analyses. The hypothesised relationship between subject matching and retention rests on the idea that prior subject knowledge provides students with an advantage in HE progression. It is likely that academic staff will tailor curriculum and pace of instruction to the assumed knowledge base of students. Thus, they will assume that entrants to a chemistry course will have the knowledge expected of those having taken chemistry at Higher (Scotland) or Leaving Certificate (Ireland) level. In order to test whether matching provides an advantage, we need to be sure that entrants have covered the same material in their upper secondary course. Thus, we confine our analyses to those who have taken the Leaving Certificate in Ireland and Scottish-domiciled students who have taken Highers. Our final sample consists of 26,816 individuals in Ireland and 14,600^5^ individuals in Scotland.

### Measures and methods

Our dependent variable is non-continuation (‘drop-out’), defined as those who were no longer on the course one year after entry and did not transfer to another HE institution or who were not repeating. Official statistics and previous research show that the first year of study is a crucial year: students are most likely to leave HE during this year than later in their studies (OECD 2019; Kadar-Satat et al., 2016; Pigott & Frawley, 2019; Smith & Naylor, 2001).

Two aspects of HE students’ academic record in upper secondary education were examined: (1) the degree of matching between the secondary subjects taken at school and the current HE course and (2) the overall exam grades achieved. For our matching variable, we construct a variable which distinguishes between ‘0’ no matching, ‘1’ one matched subject, and ‘2’ two or more matched subjects. Analyses were conducted on the number of subjects taken in four broad areas of study: cultural subjects (for example, history and geography); foreign languages; business studies (e.g. management, accountancy or book-keeping); and sciences. Maths is compulsory in Ireland and is not counted in the matched subjects. However, in Scotland taking Maths for the Highers is optional, so it is counted as a matched subject for science-related fields.

Subjects taken at upper secondary were then matched with ISCED field of study in HE. There are 11 comparable fields of study in the two datasets: “Social studies”, “Business”, “Law”, “Arts/humanities”, “Languages”, “Education”, “Science, agricultural science and veterinary science”, “Healthcare”, “Engineering and construction”, “Computer science” and “Combined and other disciplines”. Whether or not someone took cultural studies is considered directly relevant for entry into arts and humanities (see Table S1 in the Supplementary information for a full description of the matching used). For business subjects or languages, there was also a direct match with business- and language-related fields of study at the higher level. Other secondary subjects are relevant for entry into several fields; for example, whether or not someone took science subjects is not only relevant for entry into science but also for entry into the field of computer science, heath-related and medical fields as well as engineering. We included a category ‘not applicable’ for fields at the HE level that did not directly correspond with subjects at secondary level. For example, law has no direct equivalent at secondary level.

The second dimension of upper secondary education examined was overall grades. The total UCAS score (Scotland) and Leaving Certificate points (Ireland) are taken as measures of overall school performance. UCAS has a tariff system which allows students’ grades and level of qualification to be converted into points. Since 2006 UCAS provides tariff scores for the Irish Leaving Certificate which we use here for comparability. We standardise the scores to have a mean of zero and a standard deviation of one for the sake of comparison and divide scores into quintiles.

Other individual and family factors included in our analysis are: parental social class of origin, gender, age and student nationality. For Scotland, we use the NS-SeC (National Socio-Economic) measure for origin (Rose et al., 2005). For Ireland, we use a social class measure for both father and mother based on the measure used for the Irish Census of Population. We use the dominance principle and construct the highest social class among parents and harmonize the NS-SeC measure with the Irish class measure. Due to some differences in the detailed breakdown of the class groups, we aggregate them into three categories — working class, intermediate class, service class — and we add a category for those whose information on social class is missing.

In order to take institutional differentiation into account, we include the type of HE institution a student attends in the analysis. In both countries, the degree of selectivity and hence the profile of students has been found to vary by type of HE institutions (Iannelli et al., 2011; McCoy & Smyth, 2011). For this reason, in Scotland, we differentiate between Ancient Universities, Old Universities, New Universities and other universities. In Ireland, we distinguish between Universities, Institutes of Technology, and other designated institutions (such as art colleges and colleges of education).

Descriptive analyses are followed by a series of logistic regression models conducted separately on the Scottish and Irish data. Pooled analyses are used to look at the overall effect of subject matching and grades on non-continuation before conducting separate analyses for each field of study to look at the specificities of subject matching at a finer level of detail. Results are reported in terms of average marginal effects (Mood, 2010) to allow for a comparison of patterns in Ireland and Scotland. The coefficients of the full models and the model fit statistics are reported in the Supplementary information in Tables S2 and S3.^6^

## Descriptive results

The characteristics of the Irish and Scottish samples are presented in Table 1. About 51% and 58% of students in the Irish and Scottish HE samples are women. Moreover, Scottish students are markedly younger than their Irish counterparts, reflecting institutional differences between the two countries. In Scotland, students can leave secondary school at the end of the fifth year (at age 17, skipping the final year of school) and enter HE if they have achieved the number and types of Highers needed to gain access to university. In the two samples, only a very small proportion of HE students are from a nationality other than Irish or British.

**Table 1:** Descriptive statistics of the sample characteristics (column percentages)

		*Ireland*	*Scotland*
*Sex*	Female	51	58
	Male	49	42
*Age on entry*	Under 18	8	35
	18	44	55
	19	39	8
	20	7	2
	21	3	1
*Nationality*	Ireland	96	
	Non-Irish	4	
	UK		98
	Non-UK		2
*Parental social class*	Service class	24	52
	Intermediate	37	22
	Working class	10	12
	Unknown/missing	29	14
*Irish HE institutions*	Universities	56	
	Institutes of Technology	40	
	Other	5	
*Scottish HE institutions*	Ancient Universities		32
	Old Universities		28
	New Universities		37
	Other		2
*Subject matching*	No matching	7	7
	One matching	38	14
	Two + matching	24	59
	Not applicable	30	20
*Grades - Tariff quintiles*	Lowest	18	20
	2nd lowest	17	20
	Middle tariff	18	20
	2nd highest	16	22
	Highest	17	18
	Missing tariff scores	14	1
*Total no. of cases*		26,816	14,600

Percentages do not always add up to 100% due to rounding. In the case of the Scottish data, also the total number of cases is rounded in accordance with the HESA regulations.

There is a marked difference between the two countries in terms of the origin class of HE entrants, with just over half of all HE entrants in our Scottish sample coming from a service-class background compared to approximately one fourth of Irish entrants. However, the proportion of students from intermediate backgrounds is greater in the Irish case (37% compared to 22%) and there is also a larger share of Irish entrants coming from the unknown/missing category (29% compared to 14%).

The majority of HE students in our samples attend a university in Ireland (56%) and an ancient or old university in Scotland (60%). The importance of having taken specific subjects for HE entry in Scotland is reflected in the between-country differences in subject matching, with Scottish entrants more than twice as likely to have taken two or more related subjects at secondary level (59% having done so compared with 24% in Ireland).

**Fig. 1:**
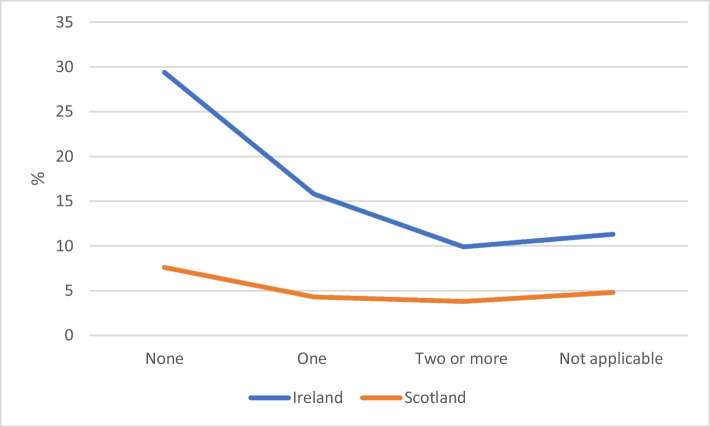
Non-continuation rates by subject matching in Ireland and Scotland

We hypothesised that, on the basis of differences in HE selection criteria, rates of non-continuation would be higher in Ireland than Scotland. This is confirmed, with approximately 4% of first-year students dropping out in Scotland in the academic year 2012–2013 compared to 14% in Ireland in 2011–2012. However, this between-country difference holds even taking account of the extent of subject matching (see Figure 1). At any given level of matching, drop-out levels are higher in Ireland than in Scotland. In both countries, drop-out is greater among those with no matched subjects and lowest among those with two or more matched subjects and those for whom matching is not applicable (because of no related secondary-level subjects). The result concerning ‘not applicable subject matching’ is very interesting since it suggests that in fields of study where student selection is not based on their prior subject knowledge, students have drop-out chances as low as students in fields of study where having two matching subjects is a significant advantage for retention.

**Fig. 2:**
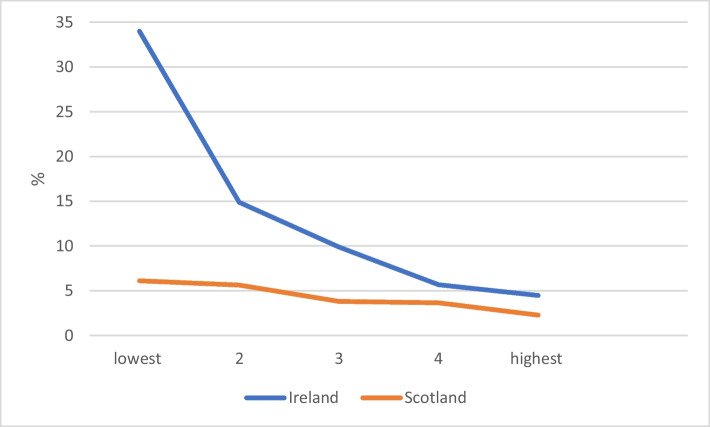
Non-continuation rates by upper-secondary grade quintile in Ireland and Scotland

Finally, drop-out rates vary significantly by upper secondary grades in both countries (Figure 2). However, there are very large differences between Ireland and Scotland in these rates for lower-achieving entrants, with moderate differences for the middle-achievement and very few differences among high-achieving entrants. The relative influence of subject matching and grades in the two national contexts is explored in the next section.

## Multivariate analyses

A series of binary logistic regression models is used to look at the effects of individual and family variables, subject matching and grades on drop-out in the two systems. Model 1 in Tables 2 and 3 focuses on social class differences, after taking into account students’ gender, age and nationality. In Ireland, significant differences in drop-out rates are evident by social class: after controlling for other individual characteristics, students with parents in working (or unknown) and intermediate classes have respectively four and three percentage points higher chances of dropping-out than those with parents in the service class. No such class difference is found in Scotland, where students from various social backgrounds show similar chances of dropping-out. This is an important result which confirms our third hypothesis and suggests that the tighter selection criteria for entering HE in Scotland may be behind the absence of social inequalities in drop-out chances found here.

**Table 2:** The probability of non-continuation — Ireland (Average Marginal Effects)

	Model 1		Model 2	Model 3	Model 4
*Social class origin* (ref: Service)					
Intermediate	0.025***		0.020***	−0.002	−0.002
	(0.005)		(0.005)	(0.006)	(0.006)
Working-class	0.033***		0.024**	−0.012	−0.015*
	(0.008)		(0.008)	(0.007)	(0.007)
Class unknown	0.035***		0.031***	0.009	0.009
	(0.006)		(0.006)	(0.006)	(0.006)
*Subject matching* (ref: one subject match)					
No matched subjects			0.112***	0.057***	0.054***
			(0.011)	(0.008)	(0.008)
2 matched subjects			−0.054***	−0.022***	−0.016**
			(0.005)	(0.005)	(0.005)
Matching not applicable			−0.038***	−0.019***	−0.013**
			(0.005)	(0.005)	(0.005)
*Grades* (ref: Highest scores)					
Lowest				0.264***	0.219***
				(0.008)	(0.009)
2^nd^ lowest				0.097***	0.083***
				(0.006)	(0.007)
Middle				0.051***	0.047***
				(0.006)	(0.006)
2^nd^ highest				0.010*	0.010
				(0.005)	(0.006)
No score				0.084***	0.078***
				(0.007)	(0.007)
*HE Institutions* (Ref: Institute of Technology)					
University					−0.039***
					(0.006)
Other					−0.116***
					(0.008)
*McFadden’s Pseudo-R*^2^		*0.017*	*0.034*	*0.106*	*0.112*
*Total number of cases*		26,816	26,816	26,816	26,816

All models control for gender, age and nationality; standard errors are in parentheses

*** *p* < 0.001, ** *p* < 0.01, * *p* < 0.05

**Table 3:** The probability of non-continuation — Scotland (Average Marginal Effects)

	Model 1	Model 2	Model 3	Model 4
*Social class origin* (ref: Service)				
Intermediate	−0.003	−0.003	−0.005	−0.005
	(0.004)	(0.004)	(0.004)	(0.004)
Working-class	0.004	0.004	0.0006	0.0009
	(0.006)	(0.006)	(0.005)	(0.005)
Class unknown	0.004	0.004	0.003	0.004
	(0.005)	(0.005)	(0.005)	(0.005)
*Subject matching* (re: one subject match)				
No matched subjects		0.032***	0.026**	0.027**
		(0.010)	(0.009)	(0.009)
2 matched subjects		−0.006	−0.001	−0.003
		(0.005)	(0.004)	(0.005)
Matching not applicable		0.005	0.009	0.007
		(0.006)	(0.006)	(0.006)
*Grades* (ref: Highest scores)				
Lowest			0.035***	0.044***
			(0.005)	(0.006)
2^nd^ lowest			0.032***	0.036***
			(0.005)	(0.005)
Middle			0.015***	0.016***
			(0.005)	(0.005)
2^nd^ highest			0.014***	0.013**
			(0.005)	(0.004)
No score			0.060	0.076
			(0.082)	(0.094)
*Institutions* (Ref: Ancient universities)				
Old universities				0.003
				(0.005)
New universities				−0.013**
				(0.005)
Other universities				−0.036***
				(0.008)
*McFadden’s Pseudo-R*^2^	*0.005*	*0.011*	*0.021*	*0.026*
*Total number of cases*	14,600	14,600	14,600	14,600

All models control for gender, age and nationality; standard errors are in parentheses

*** *p* < 0.001, ** *p* < 0.01, * *p* < 0.05

Model 2 adds in subject matching. In both countries, higher drop-out chances are found among those who had taken no matched subjects. Moreover, in Ireland, drop-out chances are significantly lower among those who have two or more matched subjects than among students with one matched subject. In line with hypothesis two, the effect of subject matching is stronger in Ireland than in Scotland. It is worth noting though that we found that rates of dropping out are higher in Ireland at any level of subject matching. After controlling for student characteristics, students who entered fields where ‘subject matching is not applicable’ have significantly lower chances of dropping out than students with one subject matching in Ireland only. Finally, lower grades are associated with much greater chances of drop-out in both countries (model 3); again, the size of the effect is greater in Ireland than in Scotland.

The initial influence of social class background on drop-out in Ireland is found to be mediated by between-class differences in subject matching: in this country, subject matching explains between 13 and 26% of the social class gap and subjects and grades together explain almost all the gap. The effect of matching on drop-out is halved in magnitude when grades are taken into account, indicating an overlap between high grades and taking relevant subjects.

Model 4 shows significant variation in drop-out by type of institution. All else being equal, non-continuation chances are highest in the institutes of technology and lowest in the universities in Ireland. This result holds both when examining raw percentages (Table 4) and the results from the logistic regression modelling which controls for individual and family factors, grades and subject matching (Table 2, model 4). In Scotland, the situation is more complex. Descriptive statistics show lower rates of student drop-out in ancient and other universities than in old and new universities (Table 4). However, in model 4 of Table 3, after controlling for students’ characteristics including subject matching and grades, students from ancient universities (together with students from old universities) have higher chances of dropping-out than students in new and other universities. This indicates that the lower overall drop-out rates of students attending research-intensive universities in Scotland are due to their student composition since, when selecting applicants, these universities put a strong emphasis on ensuring the best fit between student knowledge and ability and the study programme.

**Table 4:** Retention by HE Institution, Ireland and Scotland (row percentages)

	*Continuing*	*Non continuing*
*Irish HE institutions*		
Institutes of Technology	76	24
Universities	92	8
Other	97	3
*Scottish HE institutions*		
Ancient Universities	96	4
Old universities	95	5
New Universities	95	5
Other Universities	99	1

Drop-out rates vary by field of study with different patterning in the two countries (see Table S4 in the Supplementary information). In addition, some fields require more matched subjects and/or higher grades. Therefore, to investigate whether our findings could be driven by particular fields, we further decompose our results by running the same analysis separately in each of the eight fields of study where relevant secondary school subjects could be matched (Tables S5 and S6 in the Supplementary information). The results suggest that, after controlling for student characteristics including school grades, lack of subject preparedness (‘no subject matching’) is generally associated with higher chances of dropping out in all fields in Ireland and in most fields in Scotland. However, the estimates of having no matched subjects are significant only for students taking engineering or computing programmes in Ireland and for students studying business in Scotland. Having taken two or more matched subjects significantly enhances retention in science and business in Ireland, suggesting that the finding related to the lower chances of dropping out among those who entered HE with ‘two or more matched subjects’ (in Table 2) is mostly driven by these two fields.

## Conclusions

There is now a large body of research documenting the role of individual, family background, and institutional factors in shaping HE retention. However, much less attention has been paid to the way in which macro-level factors, in particular the way in which national admission policies and curriculum continuity from school to HE, may shape the level and nature of drop-out. This article draws on student records data to provide new insights into the role of selection criteria using two contrasting cases, Ireland where selection into HE is largely a centralized process, determined by students’ overall grades, and Scotland where HE institutions, especially the research-intensive universities, select their applicants based on grades and types of subjects taken at upper secondary level. We expected that these system differences matter in shaping levels and patterns of student drop-out in the two countries.

Our findings show that, in both Ireland and Scotland, the patterns are as expected, with higher drop-out rates among those who had not taken relevant subjects and had achieved lower grades at secondary level. Given the importance of ‘facilitating’ subjects in access to Scottish HE, it was hypothesised that overall levels of drop-out will be lower in Scotland than Ireland, due to the strong emphasis on subject preparedness that universities place in the selection of Scottish HE entrants (hypothesis 1). The effect of subject matching on retention was expected to be stronger in Ireland as non-matched candidates will mostly have been filtered out by the Scottish admission system (hypothesis 2). Much lower drop-out rates are indeed found in Scotland. Moreover, the association between subject matching and the chances of dropping-out is stronger in Ireland than in Scotland. The effect of entering HE with lower exam grades is also found to be stronger in Ireland than in Scotland, with very large between-country differences in drop-out rates among lower-achieving entrants.

In both countries, students attending different HE institutions vary in their chances of dropping-out, with the institutes of technology in Ireland and the new universities in Scotland having the highest drop-out rates. This is likely to be due to their less stringent entry criteria than other HE institutions. Indeed, when controlling for student characteristics, school grades and subject matching, we found that the gap in drop-out between students from research-intensive institutions and the other institutions largely reduces in Ireland and even reverses in Scotland. This highlights that admissions criteria are important not only to explain between country differences in drop-out rates but also within-country differences.

Finally, we hypothesised that social inequalities in the chances of dropping out would be lower in Scotland than in Ireland (hypothesis 3). The results of our analysis show that there are no significant differences between students from different social origins in Scotland while there are significant differences in Ireland. These differences reduce when school subjects and grades are included in the modelling, indicating that a lower academic preparedness may be behind the higher drop-out chances of less socially advantaged students in Ireland.

These results provide new evidence on the importance of admissions policies and curriculum continuity for HE retention. In summary, in Scotland strict HE selection criteria based on students’ school attainment and curriculum ensure high student retention rates. This is because students with higher grades on entry are better able to cope with complex course material while those who have studied subjects at school level closely related to the field of study entered have the foundational knowledge to better engage with their tertiary course. However, the drawback of such a system is that students from disadvantaged social backgrounds are less likely to meet those strict entry criteria (Iannelli, Smyth & Klein, 2016), thus ending up being excluded from entering HE altogether or from attending the most prestigious universities or certain disciplines which have been found to lead to better occupational returns (Belfield et al., 2018).

In contrast, when prior knowledge in specific subjects is not required as in Ireland, students may find it easier to gain access to HE and to different fields of study but they may find it harder to progress having entered. This may be particularly the case if lecturers do not take into account that students may have varied levels of knowledge of the subject taught and they do not adjust the course level and material to allow a smoother transition to university. Thus, the drawback of this more open system is delayed student selection which involves a waste of resources for both students and institutions.

From a policy perspective, the issue is: how can HE systems support widening access and at the same time ensure that all students from whatever background have high chances of succeeding?

It is clear that there is a need to strike a balance between selecting students who are likely to succeed once they have entered HE and promoting equality of opportunity by allowing students from different social backgrounds and with different prior knowledge equal chances of entering HE. Improving retention then will require targeted academic support, particularly in the first year of study, for those students who lack a strong academic, subject-related background at entry.

HE institutions in different countries have introduced different forms of intervention to improve student retention, e.g. financial support, peer mentoring, pre-university and first-year remedial courses, guidance and counselling services. The available evidence on the effectiveness of these initiatives is limited and often descriptive. Those studies, which provide robust evidence based on experimental and quasi-experimental designs or causal inference methods, report positive effects of interventions which tackle multiple aspects (financial, academic and social aspects) of student HE experiences (Denny et al., 2014; Angrist et al., 2009) or target subject-specific deficit (Lesik, 2007). More research of this kind is required to establish the most effective policies and practices and investigate whether they can be scaled up at national level. However, the focus of these interventions should not be confined to HE. A system-wide approach to the problem of HE retention is needed, which recognises the importance of reducing social inequalities in student achievement and subject choices at school and of improving the exchange of information between schools and universities to promote smooth progression in student learning.

## Supplementary Information

Below is the link to the electronic supplementary material.

Supplementary file1 (DOCX 39 KB)

## Data Availability

The study used customised linked administrative data provided by the Higher Education Authority in Ireland and the Higher Education Statistics Agency in the UK. Access to the data is at the discretion of the two Authorities and was provided to the research team for the purpose of the project. The authors cannot share the datasets with anybody outside the project.

## Footnotes

1 In addition, there are 15 private HE institutions, with over 15,000 students. These institutions are not included in official statistics and so rates of non-progression cannot be analysed for this group. Since the period covered by the data, a number of institutes of technology have formed technological universities.

2 HE courses (at sub-degree level) are also offered in Scottish further education colleges. However, these students are not included in Higher Education Statistics Agency (HESA) data and so are not analysed in this paper.

3 This online tool can be accessed at: https://www.informedchoices.ac.uk/

4 The distinction between being new to HE-level courses and being new to the HE institution sector is an important one in the Scottish context. Many students have taken a sub-degree qualification at a further education college and thus would not be considered new entrants to HE.

5 The total number of cases for Scotland has been rounded to the nearest figure in accordance with the HESA regulations.

6 Following Rubin (1985), we report the statistical significance of the parameters to test whether our independent variables are better able to explain the observed sub-population differences than chance. Moreover, our single cohort of students can be thought of as a sample of the larger population of first year students who are at university at different times.
