# Supplementary material for: Higher education retention in Ireland and Scotland: the role of admissions policies
Source: High Educ (Dordr). 2024 Jul 11;89(4):1021–39. doi: 10.1007/s10734-024-01259-1 (PMC12045830; doi:10.1007/s10734-024-01259-1)
Supplement: Supplementary file 1 — Supplementary file1 (DOCX 39 KB) [file 10734_2024_1259_MOESM1_ESM.docx]

**Supplementary material**

**Table S1: Matching of HE ISCED Fields of Study and upper secondary subject groups**

| HE Field of Study | Upper secondary matched groups |
| --- | --- |
| Education | Not applicable |
| Arts and Humanities | Number of cultural subjects (e.g. History, Geography, Art, Music and Drama) |
| Languages | Number of languages |
| Social Studies | Not applicable |
| Business | Number of business subjects |
| Law | Not applicable |
| Computer science & Math science | Number of science subjects (or math or computer science) |
| Engineering and Construction | Number of science subjects (or math or computer science) |
| Medicine & Veterinary | Number of science subjects (or math or computer science) |
| Sciences, Agriculture | Number of science subjects (or math or computer science) |
| Healthcare related | Number of science subjects (or math or computer science) |
| Combined and other disciplines / Basic/general programmes | Not applicable |

Notes: matched subjects coded as 0 (‘None’), 1, 2 (‘2 or more’) and ‘not applicable’ (i.e. there is no relevant matched subject at upper secondary level).

| **Table S2: The probability of non-continuation - Ireland (Coefficients)** | | | | | | | | | | | | | |  |
| --- | --- | --- | --- | --- | --- | --- | --- | --- | --- | --- | --- | --- | --- | --- |
|  | Model 1 | | | Model 2 | | | | | Model 3 | | | Model 4 | | |
| *Constant* | -1.890***  (0.047) | | | -1.762***  (0.052) | | | | | -2.845***  (0.086) | | | -2.500***  (0.097) | | |
| *Social class origin*  (ref: Service) |  | | |  | | | | |  | | |  | | |
| Intermediate | 0.226*** | | | 0.182*** | | | | | -0.016 | | | -0.023 | | |
|  | (0.049) | | | (0.050) | | | | | (0.052) | | | (0.052) | | |
| Working-class | 0.287*** | | | 0.219** | | | | | -0.118 | | | -0.142* | | |
|  | (0.067) | | | (0.068) | | | | | (0.071) | | | (0.071) | | |
| Class unknown | 0.310*** | | | 0.276*** | | | | | 0.078 | | | 0.078 | | |
|  | (0.050) | | | (0.051) | | | | | (0.053) | | | (0.053) | | |
| *Female* | -0.503*** | | | -0.409*** | | | | | -0.246*** | | | -0.227*** | | |
|  | (0.036) | | | (0.037) | | | | | (0.039) | | | (0.039) | | |
| *Age* (ref: 18) |  | | |  | | | | |  | | |  | | |
| Under 18 | 0.074 | | | 0.051 | | | | | -0.080 | | | -0.073 | | |
|  | (0.069) | | | (0.070) | | | | | (0.072) | | | (0.073) | | |
| 19 years | 0.045 | | | 0.051 | | | | | 0.063 | | | 0.054 | | |
|  | (0.040) | | | (0.040) | | | | | (0.042) | | | (0.042) | | |
| 20 years | 0.508*** | | | 0.464*** | | | | | 0.291*** | | | 0.230*** | | |
|  | (0.064) | | | (0.065) | | | | | (0.068) | | | (0.068) | | |
| 21 years | 0.690*** | | | 0.612*** | | | | | 0.407*** | | | 0.321** | | |
|  | (0.095) | | | (0.096) | | | | | (0.100) | | | (0.100) | | |
| *Non-Irish* | 0.244*** | | | 0.240** | | | | | 0.008 | | | -0.019 | | |
|  | (0.080) | | | (0.081) | | | | | (0.084) | | | (0.084) | | |
| *Subject matching*  (ref. one subject match) |  | | |  | | | |  | | |  | | | |
| No matched subjects |  | | | 0.695*** | | | | | 0.445*** | | | 0.433*** | | |
|  |  | | | (0.058) | | | | | (0.061) | | | (0.061) | | |
| 2 matched subjects |  | | | -0.499*** | | | | | -0.208*** | | | -0.153** | | |
|  |  | | | (0.050) | | | | | (0.052) | | | (0.053) | | |
| Matching not applicable |  | | | -0.330*** | | | | | -0.177*** | | | -0.125** | | |
|  |  | | | (0.045) | | | | | (0.047) | | | (0.047) | | |
| *Grades* (ref: Highest scores) | | | | | | | | | | | | | | |
| Lowest |  | | |  | | | | | 2.196*** | | | 1.881*** | | |
|  |  | | |  | | | | | (0.080) | | | (0.091) | | |
| 2^nd^ lowest |  | | |  | | | | | 1.207*** | | | 1.010*** | | |
|  |  | | |  | | | | | (0.084) | | | (0.088) | | |
| Middle |  | | |  | | | | | 0.773*** | | | 0.662*** | | |
|  |  | | |  | | | | | (0.087) | | | (0.088) | | |
| 2^nd^ highest |  | | |  | | | | | 0.200* | | | 0.174 | | |
|  |  | | |  | | | | | (0.097) | | | (0.097) | | |
| No score |  | | |  | | | | | 1.091*** | | | 0.965*** | | |
|  |  | | |  | | | | | (0.088) | | | (0.092) | | |
| *HE Institutions* (Ref: IoT) | | | | | | | | | | | | | | |
| University | |  | | | |  | | | |  | | | -0.351*** | |
|  | |  | | | |  |  | | | | | | (0.050) | |
| Other | |  |  | | | | | | |  | | | -1.497***  (0.175) | |
| LR (df=9, 12, 17, 19) | 369.277*** | | | | 734.058*** | | | | | 2298.272*** | | | 2432.880*** | |
| McFadden’s Pseudo-R^2^ | 0.017 | | | | 0.034 | | | | | 0.106 | | | 0.112 | |
| BIC (df=10, 13, 18, 20) | 21410.845 | | | | 21076.654 | | | | | 19563.424 | | | 19449.210 | |
| *Total number of cases* | 26,816 | | | | 26,816 | | | | | 26,816 | | | 26,816 | |
| Notes: Standard errors in parentheses  *** p<0.001, ** p<0.01, * p<0.05 | | | | | | | | | | | | | |  |

**Table S3: The probability of non-continuation - Scotland (Coefficients)**

|  | Model 1 | | Model 2 | | | Model 3 | | | | Model 4 |  |  |
| --- | --- | --- | --- | --- | --- | --- | --- | --- | --- | --- | --- | --- |
| *Constant* | -2.973*** | | -2.932*** | | | -3.608*** | | | | -3.565*** |  |  |
|  | (0.079) | | (0.132) | | | (0.185) | | | | (0.190) |  |  |
| *Social class origin* (ref: Service) |  |  | | |  | | |  | | | |  |
| Intermediate | -0.078 | | -0.079 | | | -0.133 | | | | -0.120 |  |  |
|  | (0.108) | | (0.108) | | | (0.108) | | | | (0.108) |  |  |
| Working-class | 0.094 | | 0.101 | | | 0.015 | | | | 0.022 |  |  |
|  | (0.128) | | (0.128) | | | (0.129) | | | | (0.129) |  |  |
| Class unknown | 0.105 | | 0.116 | | | 0.071 | | | | 0.085 |  |  |
|  | (0.119) | | (0.119) | | | (0.120) | | | | (0.120) |  |  |
| *Female* | -0.211** | | -0.256** | | | -0.234** | | | | -0.233** |  |  |
|  | (0.082) | | (0.083) | | | (0.083) | | | | (0.083) |  |  |
| *Age* (ref: 18) |  | |  | | |  | | | |  |  |  |
| Under 18 | -0.173 | | -0.171 | | | -0.180 | | | | -0.184* |  |  |
|  | (0.092) | | (0.092) | | | (0.093) | | | | (0.093) |  |  |
| 19 years of age | 0.335* | | 0.285* | | | 0.206 | | | | 0.199 |  |  |
|  | (0.136) | | (0.137) | | | (0.137) | | | | (0.138) |  |  |
| 20 years of age | -0.076 | | -0.184 | | | -0.320 | | | | -0.306 |  |  |
|  | (0.313) | | (0.315) | | | (0.316) | | | | (0.317) |  |  |
| 21 years of age | 0.684* | | 0.606* | | | 0.432 | | | | 0.403 |  |  |
|  | (0.296) | | (0.297) | | | (0.299) | | | | (0.300) |  |  |
| *Non-UK* | -0.051 | | -0.023 | | | -0.023 | | | | -0.032 |  |  |
|  | (0.278) | | (0.279) | | | (0.279) | | | | (0.280) |  |  |
| *Subject matching*  (ref: one subject match) |  |  | |  | | | | |  | | | |
| No matched subjects |  | | 0.580*** | | | 0.529** | | | | 0.540** |  |  |
|  |  | | (0.165) | | | (0.165) | | | | (0.166) |  |  |
| 2 matched subjects |  | | -0.160 | | | -0.036 | | | | -0.070 |  |  |
|  |  | | (0.125) | | | (0.127) | | | | (0.127) |  |  |
| Matching not applicable |  | | 0.122 | | | 0.218 | | | | 0.169 |  |  |
|  |  | | (0.140) | | | (0.141) | | | | (0.142) |  |  |
| *Grades* (ref: Highest scores) |  |  | |  | | | | |  | | | |
| Lowest |  | |  | | | 0.941*** | | | | 1.166*** |  |  |
|  |  | |  | | | (0.154) | | | | (0.168) |  |  |
| 2^nd^ lowest |  | |  | | | 0.887*** | | | | 1.014*** |  |  |
|  |  | |  | | | (0.154) | | | | (0.160) |  |  |
| Middle |  | |  | | | 0.509*** | | | | 0.577*** |  |  |
|  |  | |  | | | (0.162) | | | | (0.165) |  |  |
| 2^nd^ highest |  | |  | | | 0.471** | | | | 0.494** |  |  |
|  |  | |  | | | (0.161) | | | | (0.161) |  |  |
| No score |  | |  | | | 1.326 | | | | 1.601 |  |  |
|  |  | |  | | | (1.083) | | | | (1.088) |  |  |
| *Institutions*  (Ref: Ancient universities) |  |  | |  | | | | |  | | | |
| Old universities |  | |  | | |  | | | | 0.072 |  |  |
|  |  | |  | | |  | | | | (0.108) |  |  |
| New universities |  | |  | | |  | | | | -0.326** |  |  |
|  |  | |  | | |  | | | | (0.115) |  |  |
| Other universities |  | |  | | |  | | | | -1.370** |  |  |
|  |  | |  | | | |  | | | (0.513) |  |  |
| LR (df=9, 12, 17, 20) | 26.040*** | | 55.258*** | | | | 109.647*** | | | 133.704*** |  |  |
| McFadden’s Pseudo-R^2^ | 0.005 | | 0.011 | | | | 0.021 | | | 0.026 |  |  |
| BIC (df=10, 13, 18, 21) | 5268.622 | | 5268.170 | | | | 5261.725 | | | 5266.434 |  |  |
| *Total number of cases* | 14,600 | | 14,600 | | | | 14,600 | | | 14,600 |  |  |
| Notes: Standard errors in parentheses  *** p<0.001, ** p<0.01, * p<0.05 | | | | | | | | | | |  |  |

| **Table S4: Retention by field of study in Ireland and Scotland (percentages)** | | |  |
| --- | --- | --- | --- |
| Field of Study | Ireland  Non-continuation | Scotland  Non-continuation | |
| Social studies | 9 | 4 | |
| Law | 7 | 5 | |
| Business | 19 | 5 | |
| Languages | 10 | 4 | |
| Humanities | 12 | 4 | |
| Education | 3 | 6 | |
| Medicine | 4 | 1 | |
| Science, Agricultural Science | 12 | 4 | |
| Healthcare related | 8 | 5 | |
| Engineering | 23 | 4 | |
| Computer | 24 | 6 | |
| Combined | 11 | 6 | |

| **Table S5: Probability of non-continuation in Ireland, controlling for all variables in model 4 of Table 2 (AME)** | | | | | | | | | | | | | | | |
| --- | --- | --- | --- | --- | --- | --- | --- | --- | --- | --- | --- | --- | --- | --- | --- |
|  | Business | Languages | | Humanities | | Medicine | | Science | | Health | | Engineering | Computing | | |
|  |  |  | |  | |  | |  | |  | |  |  | | |
| No match | 0.02 |  | | 0.03 | |  | | 0.05 | | 0.07 | | 0.07** | 0.10** | | |
|  | (0.02) |  | | (0.03) | |  | | (0.04) | | (0.05) | | (0.02) | (0.03) | | |
| 2+ matches | -0.05** | 0.02 | | -0.01 | | -0.04 | | -0.03* | | 0.03* | | 0.02 | 0.00 | | |
|  | (0.01) | (0.05) | | (0.01) | | (0.03) | | (0.01) | | (0.01) | | (0.02) | (0.03) | | |
| Observations | 5,540 | 174 | | 3,595 | | 518 | | 3,406 | | 2,026 | | 3,095 | 1,466 | | |
| Notes: Standard errors in parentheses, ** p<0.01, * p<0.05. | | | | | | | | | |  | |  |  | | |
|  |  |  |  |  |  |  |  |  |  |  | |  |  | | |
| **Table S6: Probability of non-continuation in Scotland, controlling for all variables in Model 4 of Table 3 (AME)** | | | | | | | | | | | | | | |  |
|  | Business | Language | Humanities | | Medicine | | Science | | Health | | Engineering | | | Computing |  |
|  |  |  |  | |  | |  | |  | |  | | |  |  |
| No match | 0.04** | -0.008 | 0.07 | | - | | 0.01 | | -0.01 | | -0.01 | | | 0.16 |  |
|  | (0.01) | (0.02) | (0.07) | | - | | (0.02) | | (0.02) | | (0.03) | | | (0.09) |  |
| 2+ matches | -0.01 | -0.001 | 0.008 | | - | | -0.009 | | -0.0006 | | 0.002 | | | 0.004 |  |
|  | (0.01) | (0.02) | (0.01) | | - | | (0.01) | | (0.02) | | (0.02) | | | (0.03) |  |
| Observations | 1,930 | 640 | 1,055 | |  | | 2,880 | | 1,560 | | 1,840 | | | 960 |  |
| Notes: Standard errors in parentheses, ** p<0.01. Entry to medicine requires two or more matching subjects, for this reason no analysis could be carried out for this field. The total number of cases are rounded according to the HESA regulations. | | | | | | | | | | | | | | |  |
